# Supplementary material for: Influenza A Virus Infection Reactivates Human Endogenous Retroviruses Associated with Modulation of Antiviral Immunity
Source: Viruses. 2022 Jul 21;14(7):1591. doi: 10.3390/v14071591 (PMC9320126; doi:10.3390/v14071591)
Supplement: Supplementary file 1 [file viruses-14-01591-s001.zip › MotifAnalysis.zip!/MotifOutput_SC35MdNS1/homerResults/motif8.similar.html]

motif8

## Information for motif8

T
A
G
C
A
C
G
T
A
G
T
C
A
C
G
T
A
C
T
G
C
A
T
G
A
C
G
T
A
C
T
G
C
T
A
G
A
C
T
G
A
C
T
G
G
T
C
A
  
Reverse Opposite:  

C
A
G
T
A
G
T
C
A
G
T
C
A
G
T
C
A
G
T
C
C
G
T
A
G
T
A
C
A
G
T
C
C
G
T
A
A
C
T
G
C
G
T
A
A
T
C
G
  

|  |  |
| --- | --- |
| p-value: | 1e-49 |
| log p-value: | -1.142e+02 |
| Information Content per bp: | 1.902 |
| Number of Target Sequences with motif | 28.0 |
| Percentage of Target Sequences with motif | 36.36% |
| Number of Background Sequences with motif | 138.1 |
| Percentage of Background Sequences with motif | 0.31% |
| Average Position of motif in Targets | 105.4 +/- 47.1bp |
| Average Position of motif in Background | 107.4 +/- 92.7bp |
| Strand Bias (log2 ratio + to - strand density) | 10.0 |
| Multiplicity (# of sites on avg that occur together) | 1.21 |
| Motif File: | file (matrix) reverse opposite |

### Similar de novo motifs found

|  |  |  |  |  |  |  |  |
| --- | --- | --- | --- | --- | --- | --- | --- |
| Rank | Match Score | Redundant Motif | P-value | log P-value | % of Targets | % of Background | Motif file |
| 1 | 0.676 | A C G T C T A G A G T C A C G T A G T C A C T G A C T G A C T G A C G T A C T G | 1e-46 | -106.360841 | 33.77% | 0.28% | motif file (matrix) |
| 2 | 0.640 | A T G C C G T A A G T C A G T C A G T C A C T G C G T A A C T G | 1e-7 | -18.023730 | 11.69% | 0.81% | motif file (matrix) |
